# Women’s perceptions of and experiences with the use of misoprostol for treatment of incomplete abortion in central Malawi: a mixed methods study

**DOI:** 10.1186/s12978-022-01549-w

**Published:** 2023-02-02

**Authors:** Bertha Magreta Chakhame, Elisabeth Darj, Mphatso Mwapasa, Ursula Kalimembe Kafulafula, Alfred Maluwa, Jon Øyvind Odland, Maria Lisa Odland

**Affiliations:** 1 Norwegian University of Science and Technology, Trondheim, Norway; 2 Kamuzu University of Health Sciences, Blantyre, Malawi; 3 Malawi University of Science and Technology, Thyolo, Malawi; 4 School of Health Systems and Public Health, Faculty of Health Sciences, University of Pretoria, Pretoria, 0002 South Africa; 5 Faculty of Biosciences and Aquaculture, Nord University, Bodø, Norway; 6 Department of Obstetrics and Gynecology, St. Olav’s University Hospital, Trondheim, Norway; 7 Malawi-Liverpool-Welcome Trust Research Institute, Blantyre, 312225 Malawi; 8 Institute of Life Course and Medical Sciences, University of Liverpool, Liverpool, L69 3BX UK

**Keywords:** Misoprostol, First trimester, Incomplete abortion, Post-abortion care

## Abstract

**Background:**

Abortion-related complications are among the common causes of maternal mortality in Malawi. Misoprostol is recommended for the treatment of first-trimester incomplete abortions but is seldom used for post-abortion care in Malawi.

**Methods:**

A descriptive cross-sectional study that used mixed methods was conducted in three hospitals in central Malawi. A survey was done on 400 women and in-depth interviews with 24 women receiving misoprostol for incomplete abortion. Convenience and purposive sampling methods were used and data were analysed using STATA 16.0 for quantitative part and thematic analysis for qualitative part.

**Results:**

From the qualitative data, three themes emerged around the following areas: experienced effects, support offered, and women’s perceptions. Most women liked misoprostol and reported that the treatment was helpful and effective in expelling retained products of conception. Quantitative data revealed that the majority of participants, 376 (94%) were satisfied with the support received, and 361 (90.3%) believed that misoprostol was better than surgical treatment. The majority of the women 364 (91%) reported they would recommend misoprostol to friends.

**Conclusions:**

The use of misoprostol for incomplete abortion in Malawi is acceptable and regarded as helpful and satisfactory among women.

## Plain language summary

One of the major causes of maternal mortality is complications after abortion and miscarriages. The importance of post-abortion care in preventing such deaths justifies the necessity of making treatment accessible and available to every woman in need. Misoprostol is one of the approved treatments for incomplete abortion but is rarely used in developing countries. A study was conducted in three hospitals in central Malawi where women filled in a questionnaire and were interviewed after receiving misoprostol for incomplete abortion. The study's goal was to investigate women’s experiences and feelings about using misoprostol. The findings showed that the majority of the women received medication and counselling as a form of support. They expressed satisfaction with the support and described misoprostol as being useful in removing retained products of conception from the womb. The medication was preferred and regarded as a reliable treatment that was also good for women. The majority of the women reported tolerable side effects of the drug and would recommend it to friends. In conclusion, the use of misoprostol for early incomplete abortion in Malawi is acceptable and is regarded as helpful, and satisfactory to women receiving post-abortion care. The research findings support expanding use of misoprostol in post-abortion care in Malawi.

## Background

Malawi, a country in Sub-Saharan Africa, has one of the highest maternal mortality ratios in the world. The current maternal mortality ratio is 439/100,000 live births [1, 2]. Maternal deaths are defined as any deaths that occur during pregnancy or childbirth, or within 42 days after birth or termination of pregnancy [2]. It is estimated that 6–7% of these deaths in Malawi are due to complications of abortions [1]. Malawi has a restrictive abortion law, and termination of pregnancy in Malawi can only be performed when the life of the woman is in danger. Attempts to change the law have been made but the bill has not yet been passed in parliament [3]. It is an offence to procure or assist a woman in procuring a miscarriage according to the laws of Malawi [4]. Because of the restrictive abortion law, women seek illegal abortions which are often done by untrained providers and/or using unsafe methods. This puts the women at a higher risk of complications such as incomplete abortion and trauma which, if left untreated, can lead to haemorrhage, sepsis and death. Incomplete abortion is a common complication after both spontaneous and induced abortions, but is more common after unsafely induced abortions, and it can be treated surgically or using misoprostol [5–8]. In some countries safe abortions are possible with the correct guidance and methods through telemedicine, however, this might not be easily accessible to poor women in rural areas and in areas where abortion is restricted [9]. The World Health Organization (WHO) recommends surgical treatment with vacuum aspiration or medical treatment with misoprostol as treatments for incomplete abortion in the first trimester [10, 11]. Manual vacuum aspiration (MVA) is the recommended surgical treatment for incomplete abortions of less than 14 weeks gestation in Malawi according to the guidelines, but its use decreased in Malawian hospitals from the year 2010 [12, 13]. The low level of use of MVA is due to challenges such as lack of equipment, resources, expertise among staff, and shortage of staff [8, 12, 14]. Contrary to the WHO recommendation, sharp curettage is still being used for post-abortion care (PAC) in early pregnancy loss in spite of the high risk of complications associated with its use, such as bleeding, perforation, and infections [8, 12, 13, 15].

Misoprostol is an alternative to MVA, as recommended by WHO [11]. Studies on the management of incomplete abortions in both low and high resource settings have shown that misoprostol, a prostaglandin E1 analogue, is equally safe and effective as MVA [13–22]. Misoprostol has been reported to have high levels of acceptability and satisfaction among women for the treatment of incomplete abortions in clinical trials [15, 16, 18–20]. Studies in Africa have also shown that most women prefer medication over vacuum aspiration [23–25]. Despite knowledge of the advantages of misoprostol in PAC, its use in Malawi has remained minimal and surgical treatment with sharp curettage is used in most cases [12, 26, 27]. The use of misoprostol for treatment of incomplete abortions in Malawian hospitals has been seen to be as low as 1.3% [5]. Reluctance among clinicians due to fear of treatment failure and a longer waiting period for treatment results to manifest have been mentioned as barriers for healthcare workers to use misoprostol [12]. While there are many studies on women´s experiences on the use of misoprostol, according to our knowledge there are a few studies on women’s experiences and perceptions of the use of misoprostol for the management of incomplete abortions in low income countries [23–25, 28] but, so far, none from Malawi. There is a need for more studies in order to establish more knowledge on women’s experiences and perceptions of the use of misoprostol in the treatment of incomplete abortions.

In view of all challenges faced in the provision of high-quality PAC services in Malawi, an intervention study was carried out in selected public hospitals to improve PAC services by increasing the use of misoprostol. Whilst changing practice is important, there is a need to understand women’s views before further implementation of misoprostol is recommended. Since women’s experiences and perceptions show the level of acceptability of the treatment in PAC, this knowledge will be helpful in guiding the health care sector in the implementation of misoprostol. The aim of this study was to explore the perceptions and experiences of women who received misoprostol for the management of incomplete abortion after an intervention study in Malawi.

## Methods

### Study design

This is a mixed methods study where quantitative and qualitative data were collected simultaneously from women receiving misoprostol for first-trimester incomplete abortion. A descriptive cross-sectional study design was used for the quantitative part of the study, and an explorative method with in-depth interviews for the qualitative part. A questionnaire and an interview guide developed in English and translated into Chichewa, a local language, were used to acquire information about women’s experiences and perceptions of misoprostol as treatment for first-trimester incomplete abortion at a one-week follow-up visit after treatment. A concurrent triangulation approach was chosen for more understanding and confirmation of the findings [29, 30].

### Setting

The study was conducted in the central region of Malawi, in gynaecological wards of three government facilities, namely: Bwaila, Salima, and Mchinji district hospitals. District hospitals act as referral centres in their respective districts and provide free medical, surgical and supportive care to patients. The care provided to women with gynaecologic problems in these facilities include, among others, comprehensive emergency care and PAC. Bwaila hospital is located in Lilongwe, which is the capital city of Malawi; the other two hospitals are located in Lilongwe’s neighbouring districts.

### Study population

All women who returned for follow up between 18th August and 7th December 2020 at the three district hospitals, after being treated with misoprostol for first trimester incomplete abortion were eligible.

### Sampling and sample sizes

Both convenience and purposive sampling methods were used. The survey was done with 400 participants altogether. We targeted 200 participants at Bwaila hospital because it is a larger facility with double the number of patients with incomplete abortions per month as compared to the other two facilities. We had 100 participants from each of the other sites.

For the qualitative part, the first 24 women of different ages and number of pregnancies who had experienced misoprostol treatment for incomplete abortion were recruited from all 3 sites. The women were a subset of those who participated in the survey. Everyone was offered an opportunity to participate until after reaching data saturation. Those who were available and had the experience of being treated with misoprostol were asked to participate.

### Inclusion and exclusion criteria

All women treated for first trimester incomplete abortion with misoprostol, and who reported for a follow-up visit at one week in the study sites were eligible and asked to take part in the study. Women who had complications prior to treatment such as severe bleeding and those who did not give their consent were excluded.

### Data collection and management

Data were collected from 18th August to 7th December 2020 using a pre-tested questionnaire with two open-ended questions and an interview guide. Data were collected to determine experiences and to explore perceptions of women who received misoprostol during their check-up visit. Data collection tools targeting the women were developed in English and translated into Chichewa. Data were collected in a local language (Chichewa) and then later translated into English. Codes were used to identify the participants. Interviews were conducted by trained research assistants (nurse/midwives) in a quiet room at the facility. The women were approached after they had been checked up, before going home. Recruitment and interviews were done on the same day. Face-to-face interviews were conducted with the women by the data collectors using interview guides. The interviews lasted for about 30 min each and were audio recorded. Data collection for the qualitative part ended after 24 interviews when no more new information was obtained; this was determined by repetitive information.

For the quantitative part, data were collected through a survey with 400 participants. Research assistants used questionnaires on Android devices. The data were collected using forms generated by CSPro v7.0™ and were synched in a Dropbox by the data collectors immediately after the interviews. The research assistants were trained in the data collection and use of the android devices prior to the interviews to ensure that they were familiar with the data collection process.

### Data analysis

**Fig. 1:**
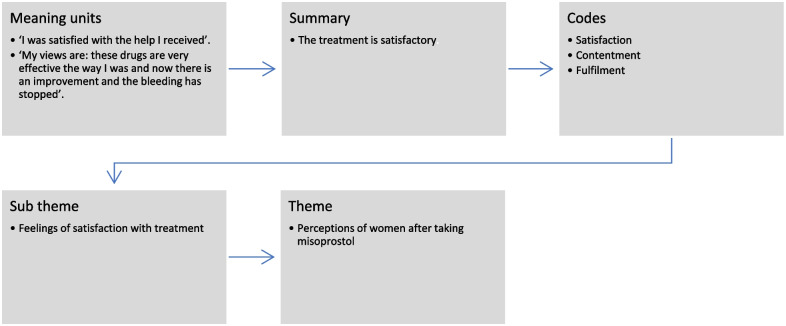
Analytical process

Quantitative data were exported from Dropbox and analysed using STATA 16.0 for detailed descriptive analyses. Descriptive statistics were computed from the demographic and other variables. The results are presented in tables and narratives. Reflexive thematic analysis using the inductive approach was used to analyse narrative data obtained from individual in-depth interviews [31]. The analysis of the qualitative data was ongoing throughout the data collection period. Transcription and translation were done immediately after each interview. The transcripts were checked after transcription and translation to ensure there was no misrepresentation of information. The scripts were read several times for familiarisation of data which was followed by the identification of codes. Coding aided organisation of the data according to the emerging concepts, then the codes were grouped under themes and sub-themes [31]. An example of the analytical process is presented in Fig. 1. The emerging themes and subthemes were revised and reported in a narrative format, illustrated by quotes, coded as responders 1 to 24.

### Ethical considerations

The study was carried out following ethical rules and guidelines. Ethical clearance was obtained from the College of Medicine Research and Ethics Committee (COMREC)—Malawi (Ref: P.01/20/2924) and Regional Committees for Medical and Health Research Ethics (REK) – Norway (Ref: 141130 2019). Permission to conduct the study in the selected sites was obtained from Lilongwe (for Bwaila), Salima and Mchinji district health offices. Written informed consent was sought prior to data collection. Participants were given information pertaining to the study through an information sheet, which was read to them. Each participant was informed of the potential risks and benefits of participating in the study and was assured of privacy and confidentiality, as no names were used for identification. The participants were informed about how their data would be managed and that only the research team would have access to the data. The women were also informed that participation in the study was voluntary and that they were free to withdraw from the study at any point. In addition, they were informed that refusal to participate or withdrawal from the study would not affect their treatment at the facility.

## Results

### Quantitative results

#### Demographic data

A total of 400 participants from the three sites were enrolled: 200 participants from Bwaila hospital in Lilongwe, and 100 participants from each of the district hospitals of Mchinji and Salima. The main demographic characteristics of the participants are shown in Table 1. The largest group of the study participants 127 (31.8%) were in the age group of 20–24 years; 176 (44%) participants had secondary education, followed by 168 (42%) who had primary education; 304 (76%) were married; 332 (83%) had their first abortion and 263 (65.8%) had a planned pregnancy.

**Table 1:** Demographic characteristics of participants n (%) treated with misoprostol for incomplete abortion in central Malawi 2020

Characteristics of women	Total population n = 400	Bwaila n = 200	Salima n = 100	Mchinji n = 100
Age in years				
15–19	64 (16.0)	24 (12.0)	19 (19.0)	21 (21.0)
20–24	127 (31.8)	60 (30.0)	37 (37.0)	30 (30.0)
25–29	92 (23.0)	60 (30.0)	16 (16.0)	16 (16.0)
30–34	74 (18.5)	34 (17.0)	18 (18.0)	22 (22.0)
35 and above	43 (10.8)	22 (11.0)	10 (10.0)	11 (11.0)
Total	400 (100)	200 (100)	100 (100)	100 (100)
Educational qualification				
Did not attend school	34 (8.5)	1 (0.5)	12 (12.0)	21 (21.0)
Primary	168 (42)	62 (31.0)	51 (51.0)	55 (55.0)
Secondary	176 (44)	119 (59.5)	34 (34.0)	23 (23.0)
Tertiary	22 (5.5)	18 (9.0)	3 (3.0)	1 (1.0)
Total	400 (100)	200 (100)	100 (100)	100 (100)
Marital status				
Single	81 (20.3)	31 (15.5)	31 (31.0)	19 (19.0)
Married	304 (76)	162 (82.5)	64 (64.0)	78 (78.0)
Divorced	7 (1.8)	2 (2.0)	2 (2.0)	3 (3.0)
Widowed	8 (2.0)	5 (2.5)	3 (3.0)	0 (0.0)
Total	400 (100)	200 (100)	100 (100)	100 (100)
Number of abortions (previous and current)				
1	332 (83)	165 (82.5)	82 (82.0)	85 (85.0)
2	59 (14.8)	29 (14.5)	15 (15.0)	15 (15.0)
3	9 (2.3)	6 (3.0)	3 (3.0)	0 (0)
Total	400 (100)	200 (100)	100 (100)	100 (100)
Planned pregnancy				
Yes	263 (65.8)	127 (63.5)	60 (60.0)	76 (76.0)
No	137 (34.3)	73 (36.5)	40 (40.0)	24 (24.0)
Total	400 (100)	200 (100)	100 (100)	100 (100)

As depicted in Table 2, the majority of women 233 (58.2%) reported no side-effects from the treatment. However, 167 (41.8%) participants reported having experienced some side effects of misoprostol, such as: lower abdominal pain 76 (33.8%); diarrhoea 35 (15.6%); nausea 28 (12.4%); heavy bleeding 26 (11.6%); mild fever 19 (8.4%); high fever 17 (7.6%), and other minor problems 24 (10.7%).

**Table 2:** Side-effects experienced by women treated with misoprostol for incomplete abortions in three public hospitals in Malawi, 2020

Experience of any side-effect after treatment with misoprostol (n=400)	n	%	
Yes	167	41.8	
No	233	58.2	

Side-effects experienced	n	% of responses (n = 225)*	% of cases (n = 167)
Severe abdominal pain	76	33.8	45.0
High fever	17	7.6	10.1
Heavy bleeding	26	11.6	15.4
Low fever	19	8.4	11.2
Diarrhoea	35	15.6	20.7
Nausea	28	12.4	16.6
Other	24	10.7	14.2

*Total number of responses is 225 as people were able to give several answers to side-effect experienced

As shown in Table 3, most participants 384 (96.2%) reported that the support offered to them was helpful. In total, 249 (62.3%) of the participants were offered family planning services. Furthermore, 376 (94%) of the participants expressed satisfaction with the support they received from healthcare workers. Similarly, 374 (93.5%) participants were satisfied with misoprostol as treatment for incomplete abortions. In addition, a majority of participants, 359 (89.8%) reported to have been given clear instructions on the treatment.

**Table 3:** Support offered to women (n = 400) with incomplete abortions in 3 public hospitals in Malawi, 2020

Area	Response	n	%
Type of support offered by the healthcare workers	Medication	384	96.0
	Counselling	190	47.6
	Ultrasound scanning	53	13.3
	Total	627	156.9
Rating of the support offered	Very helpful	119	29.8
	Helpful	265	66.4
	Not helpful	15	3.8
	Total	399	100
Level of satisfaction with care received	Extremely satisfied	112	28.0
	Satisfied	264	66.0
	Dissatisfied	13	3.3
	Extremely dissatisfied	11	2.8
	Total	400	100
Offered a family planning service	Yes	249	62.3
	No	151	37.8
	Total	400	100
Time when family planning service was offered	Immediately	138	55.0
	One week after treatment	103	41.0
	Later than a week after treatment	10	4.0
	Total	251	100

Most participants, 361 (90.3%), believed that misoprostol was better for them than surgical management. In addition, when asked about their opinions on the use of misoprostol in PAC, more participants regarded misoprostol as reliable, and that it should be routinely used. Furthermore, 25.5% of the participants considered the drug to have many benefits for the woman’s health as shown in Table 4. The majority of participants 364 (91%) indicated that they would recommend the use of misoprostol for incomplete abortion to friends.

**Table 4:** Opinions of women (n = 400) after medical treatment of incomplete abortion in public hospitals in Malawi, 2020

Opinion	n (%)
Misoprostol is the most reliable treatment and should be used routinely	176 (44.0)
Misoprostol has many benefits on the woman’s health	102 (25.5)
I am undecided about my views on Misoprostol	69 (17.3)
Misoprostol has undesirable side effects that suggest the need of caution in its use	46 (11.5)
Misoprostol is dangerous and should not be used	7 (1.8)
Total	400 (100)

### Qualitative results

Three major themes emerged from the data: ‘experienced effects of misoprostol’, ‘support offered to women by healthcare workers’, and ‘perceptions of women after taking misoprostol’. The themes and subthemes are presented in Table 5.

**Table 5:** Themes and sub-themes of perceptions and experiences of women who were treated with misoprostol, 2020

Theme	Sub-themes
Experienced effects of misoprostol	Experiences after taking misoprostol Perceived benefits of misoprostol
Support offered to women by healthcare workers	Medication Advice on family planning Advice on possible drug effects and follow-up
Perceptions of women after taking misoprostol	Feelings of satisfaction with treatment Opinion on the use of misoprostol

### Experienced effects of misoprostol

The participants experienced medical treatment of abortion in different ways. They described signs and symptoms of bleeding, pain, and expulsion of the products of conception. They acknowledged the positive effects of the drug as well as treatment failure, and adverse effects.

### Experiences after taking misoprostol

After taking the treatment, women experienced different effects of the drug. Most of the women reported experiencing effects such as abdominal pain and backache after taking the drug. In addition, the majority of women experienced mild to moderate bleeding and some women reported passage of products of conception some hours after taking the drug.

“*After I placed the drugs under my tongue it took some hours then I started experiencing backache. I was feeling like labour [pains], when labour was established it took some time then later I started bleeding*” [responder 2, a widow from Lilongwe].

“*I was passing blood only but when I checked on the pad, the pad had some clots this happened after I took the drugs and also the time I went to the toilet I heard that I dropped two things but I didn’t had a clue of those things*” [responder 4, married woman from Lilongwe].

Drug side-effects are common; almost every drug has some undesirable effects. Few women experienced side-effects of misoprostol, but some experienced shivering, headache, fever, feeling cold, diarrhoea, vomiting and heart palpitations.

“*I felt cold, Shivering, headache, Fever…. I took the drugs around 2 in the afternoon, I started feeling cold, having fever, shivering, then at 3 am I had diarrhoea then I started bleeding then it was already morning of the other day*” [responder 1, single woman from Lilongwe].

Drug failure was reported by some women who took the treatment. Despite the majority reporting benefits of misoprostol, some women indicated that though misoprostol was their preferred treatment, their problems were still not resolved after treatment. Some were still experiencing abdominal pains, others were still bleeding, and others reported having foul-smelling discharges.

“*The help was adequate but am still having the problem, it’s not resolved yet… after taking the drugs I was passing out foul discharges and was still experiencing abdominal pains then I came back here. …, I have finished the drugs last week but am still having the same experience with no improvement*” [responder 1, single woman from Lilongwe].

It was revealed that the drug was not always available at the hospital pharmacy and patients were asked to buy the drug from local pharmacies, where the drugs could be of substandard quality. The women indicated that the lack of drugs in hospitals and procurement of counterfeit drugs was a possible reason for failed treatment.

“*There were no drugs here I had to buy somewhere so I think I was not given the right drugs because I stopped bleeding but now has started again*” [responder 9, single woman from Mchinji].

### Perceived benefits of misoprostol

Women in this study highlighted a number of benefits of misoprostol. The majority indicated that the treatment received at the hospital was helpful and adequate. They indicated that misoprostol addressed their needs in PAC as their uteruses were evacuated without problems. A number of women reported more benefits of misoprostol in PAC such as pain relief and prevention of infection. Many women report a lot of pain when they have an incomplete abortion. In this study, it was reported that they did not feel much pain because they experienced relief from abdominal pain after taking the treatment.

“*The treatment that I was given was perfect because they issued drugs which helped to clean up my womb and the other drugs which helped me that, my womb should not be infected …The drugs helped me in such way that I didn’t feel any pain at all*” [responder 4, married woman from Lilongwe].

People have treatment preferences when ill. When asked about their preferred treatment, most women reported that given a choice, they would prefer to be treated using misoprostol over surgical management. Even those that had no previous abortion experience preferred misoprostol. The most cited reason was fear of pain associated with a surgical procedure.

“*I feel like they should continue to use this method of drugs I have heard that other methods are so painful… The best is the drug because when it start working in the body all things come out at once*” [responder 7, married woman from Lilongwe].

Even though the majority of women preferred misoprostol, one woman indicated that she preferred surgical procedures regardless of the pain that comes along with the procedure.

“*Better surgical procedure though it is painful*” [responder 16, married woman from Salima].

### Perceptions of women after taking misoprostol

Feelings of satisfaction were noticeable as some women reported that they did not bleed a lot after receiving the drug and that there was an improvement in their condition.

“*I was satisfied with the help I received*” [responder 3, married woman from Lilongwe].

“*My views are; these drugs are very effective the way I was and now there is an improvement and the bleeding has stopped*” [responder 20, married woman from Salima].

Sentiments of dissatisfaction were also uttered by participants. Some women indicated that though they received the medication, nothing had changed; they were still facing problems and hence were not satisfied with the use of misoprostol. The treatment failure was reported by a few participants who reported back to the clinic for review after noticing that their problem was not resolved after taking the medication.

“*I… finished the drugs last week but am still having the same experience with no improvement. … I feel like I didn’t get the right treatment because I am still having abdominal problems, I would have loved if the remains of conception were expelled*” [responder 1, single woman from Lilongwe].

When asked if they would recommend the treatment to their friends with a similar condition, some women indicated that they would advise friends to choose the drug (misoprostol) as they felt that the drug is effective and easy to use.

“*… if I can find a person with the same problem I can advise her to go to the hospital so that she can receive the same treatment that I got and also my body is very light now”. … “These drugs should not stop being given so that those people who have the problem similar to mine should be helped as well*” [responder 11, single woman from Mchinji].

### Support offered by healthcare workers

#### Medication

Patients receive different forms of support from healthcare workers when they visit the hospital. When asked about the support received from healthcare workers, most women reported that they received drugs such as misoprostol, antibiotics such as doxycycline and metronidazole or amoxicillin, and analgesics such as paracetamol or ibuprofen. The women were also advised on when, how, and for how long they should take the drugs. Some were advised to take misoprostol at the hospital. Some were advised to go home before they took misoprostol, as they could start bleeding before reaching their homes because no sanitary pads were provided at the hospital.

“*When I came here I was given drugs which I placed under the tongue and they also gave me flagyl [metronidazole] and DCN [doxycycline] and did scanning, … I was told that the drugs which I was placing under the tongue, should be placed after an hour that’s all*” [responder 1, single woman from Lilongwe].

“*I was told to take those drugs which I was given all of them according to instructions and that if I will not take the drugs my womb can get infected the medical personnel will remove it and another drug was Panado*” [responder 15, married woman from Mchinji].

#### Advice on family planning

In addition to medication, the women reported that they received advice on family planning in particular, pregnancy spacing and modern family planning methods. They were advised to wait for six months before they became pregnant again. A few women cited that they never received advice on family planning:

*“They didn’t tell me anything”* [responder 18, single woman from Salima].

“*They said I should get family planning method otherwise I will get pregnant again and there are several methods orals, Injections, Norplant*” [responder 17, married woman from Salima].

#### Advice on possible drug effects and follow-up

Advice on possible side effects of the drug and when to return for follow-up visit at the clinic was also given. The women indicated that the healthcare workers supported them with information on what to anticipate after taking misoprostol. They were informed that they could experience side effects like shivering, mild fever, diarrhoea, nausea, and vomiting. Furthermore, they were advised to report back to the clinic immediately if they experience problems or after one week if they do not experience any problems.

“*Was told that I would expect nausea/ vomiting, diarrhoea, dizziness but nothing happened to me*” [responder 8, divorced woman from Mchinji].

“*I was told that I should be admitted but after I pleaded with them they gave me drugs that I should take when I get home and was told I should come back after a week for review*” [responder 9, single woman from Mchinji].

## Discussion

This study has revealed that misoprostol is helpful, acceptable, and satisfactory with tolerable side effects among women with first-trimester incomplete abortion. Though this study did not quantify the level of effectiveness, both quantitative and qualitative findings showed that misoprostol is perceived to be effective by the women who received the drug in the three Malawian public hospitals.

Our study found that the support rendered to women was helpful and satisfactory. These results are similar to what was found in Tanzania, Zimbabwe, and Uganda, where women were greatly satisfied with the PAC they received [23–25]. In Zimbabwe women preferred misoprostol over vacuum aspiration as misoprostol was perceived to be less invasive [23]. In Uganda women were equally satisfied and accepted treatment with misoprostol from both midwives and doctors [24]. In Tanzania, women were satisfied with the care they received and they felt that they were treated well [25]. In our study, from both qualitative and quantitative results, participants indicated no heavy bleeding and improvement in condition as some of the things they felt satisfied with. Some women indicated that they would opt for the treatment again as it was not painful. This showed that the women were satisfied with the treatment. Similarly, previous studies in African countries such as Senegal, Burkina Faso, Nigeria, Niger and Mauritania have indicated high levels of satisfaction as nearly all women were satisfied with the treatment [15, 16, 19, 20].

The current study found that women were supported by healthcare workers in different ways, such as counselling and medication, this is similar to what Flink-Bochacki found in Pennsylvania. In his study it was found that women felt supported by the healthcare workers offering family planning, but many women who received family planning counselling initiated the discussion themselves. In this study by Flink-Bochaki, participants expressed a strong desire and the significance of information on family planning. In addition, they wanted an interpersonal connection with the healthcare workers [28]. In Senegal, a high percentage of women believed that they were treated well by the PAC providers as 81% discussed family planning with their provider [32]. The findings in our study revealed that about a third of the participants were not offered family planning services. Correspondingly in a study done in Tanzania, it was also found that women reported poor post-abortion counselling. Extremely low levels of contraceptive provision and uptake were revealed [25]. This is a missed opportunity to increase use of family planning, as these women could possibly come back to the hospital with a similar problem. Post-abortion contraceptive counselling is associated with increased uptake of family planning methods hence could prevent subsequent abortions [28]. Healthcare workers need to dedicate more time and have in-depth conversations with the women in order to understand their needs and address them.

The treatment with misoprostol was highly acceptable by most women in the current study from both qualitative and quantitative data. These results are similar to what was found in randomised studies in some countries in Africa such as Nigeria and Kenya. It was found that the acceptability of the treatment was high and similar in misoprostol and MVA arms of the studies [16, 18]. In addition, our study revealed that most women would recommend the treatment to their friends which is similar to what was reported by Shokry et al., where 77.8% of the women would recommend the treatment to a friend [33]. This would help in the dissemination of information as more people will hear about the treatment and will start demanding medical treatment. Their demands can lead to making PAC accessible to most women and help facilitate the uptake of the drug. These results show that women were satisfied with misoprostol and its use should be considered for inclusion in hospital policies across the country.

Misoprostol like any other drug has some side effects of which most are self-limiting. Abdominal pain and diarrhoea are common side effects caused by misoprostolic acid exposure during metabolism. Shivering and fever are side effects caused by prostaglandin's effect on the hypothalamus. In addition, nausea, vomiting, headache, and constipation are also common [34]. A few women experienced some side effects after taking the drug; the commonly experienced effects were abdominal pain, diarrhoea, shivering and fever. Even though some participants experienced side effects, most of them were satisfied with the treatment, indicating that the side effects were tolerable. This is similar to what has been reported in studies in done Nigeria, Mauritania, Niger and Burkina Faso where the drug was reported to have tolerable side effects [15, 16, 20, 22].

Our findings revealed that women considered misoprostol to be effective as it was successful in evacuating the uterus. These findings are similar to what most studies have portrayed [15–17, 19–22, 35, 36]. Very high levels of effectiveness of above 90% have been reported in most studies with a few showing a slightly reduced level of effectiveness (80–89%). The results were similar despite studies being conducted in different settings such as Nigeria, Senegal and Burkina Faso just to mention a few; using different study designs; and having a variety of sample sizes. This leads to the assumption that the drug can be used at different levels of care with the same effect. Despite the results showing that the drug is perceived to be effective and safe by many women, there is still a small percentage where the treatment was not effective and this may lead to the development of complications in the women reducing levels of drug safety [26]. The reported drug failure could be because of scarcity of drugs, it was reported that drugs were not always available at the hospitals hence patients were requested to buy drugs from local pharmacies. This could lead to procurement of poor-quality drugs, the availability of poor-quality drugs was also reported by Hagen et al. who found extremely substandard misoprostol tablets in Malawi [37].

Evidence gathered from this study can be used to make a conclusion that misoprostol is perceived to be effective, satisfactory, and acceptable among women with first-trimester incomplete abortion in central Malawi. Incomplete abortion is contributing to many maternal deaths globally and in Malawi [16]. Most of these deaths are preventable and treatable; the increased use of misoprostol can help prevent the deaths by making PAC more accessible. Implementation of misoprostol for PAC would help in improving women’s health. Our study findings would inform policy on the development of strategies that would facilitate the uptake of misoprostol in the management of first-trimester incomplete abortions. We strongly recommend the use of WHO guidelines in the management of incomplete abortions where vacuum aspiration and misoprostol are recommended as treatment options of first-trimester incomplete abortions and that both should be used as standard management. The use of misoprostol will enable the Malawi government to achieve sustainable development goal number 3 which intends to improve health and reduce maternal morbidity. In addition, we also recommend both pre-and in-service training of healthcare workers on the use of misoprostol to enable them to provide the treatment that is preferred by the majority of the women. The hospital management should also ensure the availability of the drug at all times to satisfy the needs of the women by making the service accessible at all times.

The study had some strengths and limitations; one of the study's strengths was that it used mixed methods approach giving a better understanding of women’s experiences with the use of misoprostol as breadth was added to the study. The validity of the results was enhanced by triangulation of methods, which also assisted to neutralise any bias intrinsic in the researchers and a particular method [29, 30]. In addition, participants were drawn from urban and semi-urban settings which helped to avoid selection bias and enhance external validity. Furthermore, interviews were conducted individually and in Chichewa this ensured a smooth interaction with the respondents because they were able to freely express their opinions and communicate. This contributed to the study's confirmability. To increase credibility, the analytical process has been described, and themes are supported by quotes. In addition, two experienced international researchers with experience in low and medium countries were involved together with local researchers who are familiar with the context. To ensure dependability, direct quotes from respondents have been included in the narratives to demonstrate their perspectives on the treatment. The setting, methods and context have been described thoroughly to ensure transferability, allowing the reader to apply the findings to other situations. Another strength is that it is the first study to our knowledge on the experiences and perceptions of women in Malawi treated with misoprostol for incomplete abortion. This is significant because it will aid in the understanding of women's perspectives on their treatment.

However, a descriptive cross-sectional method was used; hence the results are not guaranteed to be representative. In addition, the study was done in the central part of Malawi and hence cannot be generalised to the other parts of the country. Another limitation is that the study was hospital-based and was done only in public hospitals excluding women who used misoprostol as a treatment for incomplete abortion in private facilities and outside the hospital who could have shared their rich experiences hence the conclusions of the findings cannot be generalised. If these women were included, more insight could have been gained. Furthermore, the women were interviewed after treatment which could bring bias in responses as regards relief after receiving treatment.

## Conclusions

Misoprostol has been found to be acceptable for the treatment of incomplete abortion among women in the central region of Malawi. As Malawi is struggling with a high maternal mortality ratio, it is important to make sure that every available option to curb the problem is considered. Misoprostol is a safe, effective, and acceptable option in the treatment of first-trimester incomplete abortions. Our findings indicate that the use of misoprostol should be scaled up to ensure its availability as part of PAC services. The use of training intervention programs should be considered as a means to achieve this goal.

## Data Availability

Data is available on request from the corresponding author.

## Abbreviations

COMREC: College of Medicine Research and Ethics Committee
MVA: Manual vacuum aspiration
PAC: Post-abortion care
REK: Regional Committees for Medical and Health Research Ethics
WHO: World Health Organisation
